# Complexin 2 regulates secretion of immunoglobulin in antibody‐secreting cells

**DOI:** 10.1002/iid3.276

**Published:** 2019-11-05

**Authors:** Emi Tsuru, Kohei Oryu, Ken Sawada, Makoto Nishihara, Masayuki Tsuda

**Affiliations:** ^1^ Institute for Laboratory Animal Research, Science Research Center Kochi University Kochi Japan; ^2^ Kokorono Support Center Kochi Health Sciences Center Kochi Japan; ^3^ Center for Interdisciplinary Pain Aichi Medical University Aichi Japan

**Keywords:** antibody‐secreting cells, Complexin 2, immunoblobulin, SNARE

## Abstract

**Introduction:**

Complexins (CPLXs), initially identified in neuronal presynaptic terminals, are cytoplasmic proteins that interact with the soluble *N*‐ethylmaleimide‐sensitive factor attachment protein receptors (SNARE) complex to regulate the fusion of vesicles to the plasma membrane. Although much is known about CPLX function in neuronal synaptic vesicle exocytosis, their distribution and role in immune cells are still unclear. In this study, we investigated CPLX2 knockout (KO) mice to reveal the role of CPLXs in exocytosis of lymphocytes.

**Methods:**

We examined the expression of CPLXs and SNAREs in lymphocytes. To study the effect of CPLXs on the immune system in vivo, we analyzed the immune phenotype of CPLX2 KO mice. Furthermore, antibodies secretion from the peritoneal cavity, spleen, and bone marrow cells of wild‐type (WT) and CPLX2 KO mice were determined.

**Results:**

CPLX2 was detected in B cells but not in T cells, while other CPLXs and SNAREs were expressed at a similar level in both B and T cells. To clarify the function of CPLX2 in B lymphocytes, serum concentrations of immunoglobulin G (IgG), IgA, IgM, and IgE were measured in WT and CPLX2 KO mice using enzyme‐linked immunosorbent assay. The level of IgM, which mainly consists of natural antibodies, was higher in KO mice than that in WT mice, while the levels of other antibodies were similar in both types of mice. Additionally, we found that spontaneous secretion of IgM and IgG1 was enhanced from the splenic antibody‐secreting cells (ASCs) of CPLX2 KO mice.

**Conclusion:**

Our data suggest that CPLX2 inhibits spontaneous secretion of IgM and IgG1 from splenic ASCs. This study provides new insight into the mechanism of antibody secretion of ASCs.

## 1 INTRODUCTION

Soluble *N*‐ethylmaleimide‐sensitive factor attachment protein receptors (SNAREs) play a central role in regulating membrane fusion during neurotransmitter release.^1^, ^2^ Outside the neural tissues, SNARE proteins also function in other membrane fusion processes, such as hormone secretion,^3^ the acrosome reaction,^4^ and degranulation.^5^ Several studies have shown that peripheral blood cells, such as mast cells, plasma cells, neutrophils, and lymphocytes share the secretory machinery that includes the combination of subtypes of SNAP (Qbc‐SNARE), VAMP (R‐SNARE), and Syntaxin (Qa‐SNARE).^5^, ^6^, ^7^, ^8^, ^9^

Complexins (CPLXs) are a group of proteins that regulate calcium‐triggered fusion between vesicles and the plasma membrane through interactions with the SNARE complex.^11^ CPLX has four isoforms; CPLX1 is expressed specifically in the brain, while CPLX2 is expressed in the brain and in other secretory cells.^12^, ^13^ In addition, CPLX3 and CPLX4 are specifically expressed in the retina.^14^ In neuronal synapses, CPLX was found to simultaneously suppress spontaneous fusion and activate fast calcium‐evoked fusion. CPLX binds tightly to the SNARE complex and prevents membrane fusion. After Ca^2+^ influx, a conformational change in the SNARE complex releases CPLX, allowing the fusion to progress.^11^, ^15^ CPLX1 knockout (KO) mice show a severely impaired neural phenotype, often leading to premature death or severe ataxia.^16^ In contrast, CPLX2 KO mice display a normal phenotype with a minimal abnormality in neuronal development.^17^, ^18^

CPLXs are also found in nonnervous systems, such as in sperm,^19^ pancreatic secretory cells,^20^ and peripheral mast cells.^21^ In each of these tissues, studies have shown that CPLX is involved in the process of membrane fusion. In mast cells, CPLX2 plays a role in calcium‐triggered degranulation through syntaxin 3.^21^ Although much is known about CPLX function in synaptic vesicle exocytosis, the role of CPLX in lymphocytes remains unclear.

Similar to its function in the neuronal synapse, we hypothesize that CPLXs in lymphocytes may associate with the membrane fusion machinery. We examined the expressions of messenger RNA and protein of each of the CPLX isoforms in lymphocytes. Furthermore, we tested whether CPLX2 affects the secretion of immunoglobulins using CPLX2 KO mice.

## 2 MATERIALS AND METHODS

### 2.1 Antibodies

The following monoclonal antibodies (mAbs) for cell surface staining were used for flow cytometry analyses: CD3 AlexaFluor 647 (17A2), CD5 PE (53‐7.3), CD21 APC‐Cy7 (7E9), CD23 PerCP‐Cy5.5 (B3B4), B220 BrilliantViolet 421 (RA‐6B2), Gr‐1 BrilliantViolet 510 (RB6‐8C5), and NK1.1 PE‐Cy7 (PK436), purchased from Biolegend (San Diego, CA). CD11b PE‐CF594 (M1/70), CD138 APC (281‐2), and IgM BrilliantViolet 510 (R6‐60.2) were obtained from BD Biosciences (San Diego, CA). For Western blot analysis, rabbit anti‐SNAP23 was procured from Abcam (London, UK) and rabbit anti‐alfa‐tubulin from MBL (Aichi, Japan). Mouse monoclonal anti‐CPLX2 (Lp27) and anti‐SNAP25 (Sp12) were as described elsewhere.^22^

### 2.2 Mice

C57BL/6‐Cplx2^tm1Tyag^ 23 and WT mice were generated by mating heterozygous mice maintained under specific pathogen‐free conditions at the Animal Facility of Kochi University. Male and female mice, aged 3 to 4 months, were used in the experiments. All animal experiments were performed in accordance with the Regulations for Animal Experiments at Kochi University and were approved by the Kochi University Animal Care and Use Committee (license L‐024).

### 2.3 Cell separation

Splenocytes were prepared using Lympholyte‐M (Cedarlane, Ontario, Canada). Peritoneal cavity (PerC) cells were collected by injecting 4‐mL ice‐cold D‐PBS containing 0.5% bovine serum albumin (Sigma, St Louis, MO) into the peritoneal cavity of euthanized mice. Bone marrow cells were isolated from the femur and tibia, followed by hemolysis.

Primary T and B cells were separated using EasySep mouse T and B cell isolation kits (Stemcell Technologies, Vancouver, BC, Canada) and were further validated by flow cytometry to be greater than 97% pure CD3^+^ and B220^+^ cells, respectively.

### 2.4 Reverse‐transcripton polymerase chain reaction

Total RNA was isolated using RNeasy micro (QIAGEN, Hilden, Germany) and reverse‐transcribed using PrimeScript RT reagent kit (Takara Bio Inc., Shiga, Japan). PCR was performed using Takara Ex Taq (TaKaRa), according to the manufacturer's protocol. Primer sequences are provided in Table S1.

### 2.5 Western blot analysis

Cells or tissues were lysed using RIPA (radioimmunoprecipitation assay) or T‐PER (tissue protein extraction reagent) buffer (Thermo Fisher Scientific, Waltham, MA), followed by incubation in Laemmli sample buffer (Bio‐Rad, Richmond, CA) at 95°C. Samples (10 μg/lane) were subjected to electrophoresis and resolved on 12% gels (Bio‐Rad). Proteins were blotted on PVDF membrane (Bio‐Rad) and the membrane was incubated with mAbs against CPLX2 (1:5000), SNAP23 (1:5000), SNAP25 (1:5000), and alfa‐tubulin (1:5000). Proteins were detected using a horseradish peroxidase‐conjugated secondary Ab (GE Healthcare, Buckinghamshire, UK), followed by chemiluminescence detection (ECL; Bio‐Rad) with a Chemi‐Doc XRS Plus system (Bio‐Rad).

### 2.6 Flow cytometry

Single‐cell suspensions (1 × 10^6^ cells) were stained with combinations of the following mAbs for 20 minutes, and then washed. To assess leukocyte subsets, splenocytes were stained with mAbs against B220, CD3, NK1.1, CD11b, and Gr‐1. To assess B‐cell subpopulations, BM cells were stained with mAbs against IgM, B220, and CD138, splenocytes were stained with mAbs against B220, CD21, and CD23, and PerC cells were stained with mAbs against B220, CD5, and CD11b. Stained cells were analyzed on LSRFortessa cell analyzer (BD Bioscience). Leukocyte subsets were defined as follows (Figure S2A): B cells (B220^+^), T cells (CD3^+^NK1.1^−^), natural killer cells (CD3^−^NK1.1^+^), natural killer T cells (CD3^+^NK1.1^+^), neutrophils (CD11b^+^Gr‐1^+^), and monocytes (CD11b^+^Gr‐1^−^). B‐cell subpopulations were defined as follows (Figures 3A and S2B): CD138^+^ cells of BM (IgM^+^CD138^+^B220^−^),^27^ splenic B‐1 (B220^+^CD21^lo^CD23^−^), MZ B cells (B220^+^CD21^+^CD23^−^), follicular B cells (B220^+^CD21^int^CD23^+^), B‐1a cells (B220^lo/hi^CD11b^+^CD5^+^), B‐1b cells (B220^lo/hi^CD11b^+^CD5^−^), and B2 cells (B220^lo/hi^CD11b^−^CD5^−^).

### 2.7 Enzyme‐linked immunosorbent assay

Serum samples were collected and stored at −80°C. For serum antibody concentration data, we confirmed the Cohen *d* effect size in our previous study (n = 4). Therefore, we decided on the number of data points according to the power calculation. Primary cells of PerC, spleen, and BM were cultured (1 × 10^7^ cells/mL) in RPMI 1640 (Wako, Osaka, Japan) with penicillin, streptomycin, and 10% heat‐inactivated fetal bovine serum (Thermo Fisher Scientific) at 37°C with 5% CO_2_. After 16 hours, cell culture supernatants were harvested and centrifuged at 10 000*g* for 5 minutes at 4°C to remove debris. The levels of secreted total IgG, IgM, IgE, and IgG1 were determined using a mouse ELISA quantitation kit (Bethyl, Montgomery, TX). Total IgA, IgG2b, IgG2c, and IgG3 was determined using the Ready‐SET‐Go ELISA kit (Thermo Fisher Scientific).

### 2.8 Statistical analysis

Statistical analyses of differences between groups were determined by the unpaired Student *t* test or Mann‐Whitney *U* test (*P* ≤ .05). Statistical tests were performed using GraphPad Prism 6.0. Error bars in the figures represent standard error of the means (SEM).

## 3 RESULTS

### 3.1 Expression of CPLXs and SNAREs in lymphocytes

**Figure 1:**
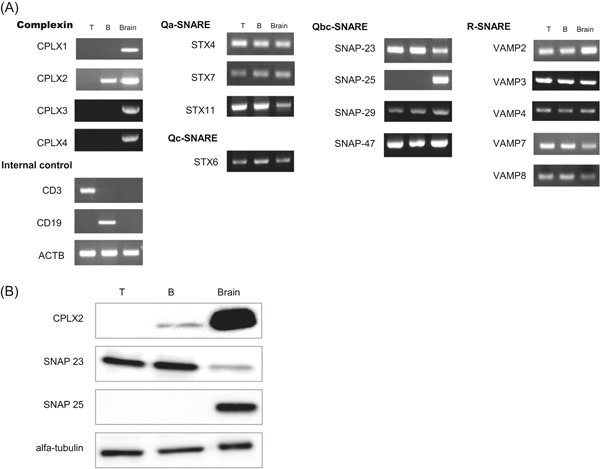
CPLX2 is expressed in B cells but not in T cells. A, CPLXs and SNAREs expressions were analyzed by RT‐PCR using total RNA from T and B cells of WT mouse spleen. Whole‐brain was used as a positive control. ACTB was used as an internal control. B, Western blot analyses show protein expression levels of CPLX2, SNAP23, and SNAP25 in T and B cells of WT mouse spleen. Alfa‐tubulin was used as a loading control. A, B, one representative data of two independent experiments with one mouse per experiment. ACTB, actin beta; CPLX, complexins; RT‐PCR, reverse‐transcription polymerase chain reaction; SNARE, soluble *N*‐ethylmaleimide‐sensitive factor attachment protein receptors; WT, wild‐type

To determine whether CPLXs were expressed in lymphocytes, we examined the expression of the four CPLX isoforms in T and B cells obtained from spleen using reverse‐transcription polymerase chain reaction (RT‐PCR). CPLX2 was expressed only in B cells, and not in T cells, although CPLX1, 3, and 4 were not detected in either T or B cells. (Figure 1A). The expression pattern of SNAREs which were reported to be expressed in immune cells was similar between B and T cells (Figure 1A). SNAP25 was expressed only in neuronal cells^22^ and not in lymphocytes (Figure 1A). Additionally, we found that the expression level of CPLX2 protein in B cells was much lower than that in the brain (Figure 1B).

### 3.2 Immunophenotype of CPLX2 KO mice

**Figure 2:**
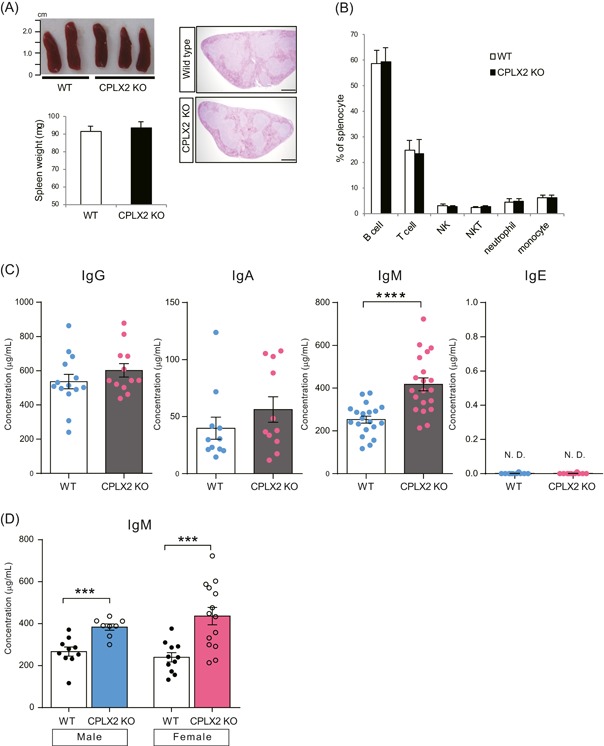
Immunophenotype analysis of CPLX2 KO mice. A, Photo of spleens, spleen weight, and H&E‐stained spleen sections (20×) of WT and CPLX2 KO mice. H&E‐stained sections are one representative image of two independent experiments with two mice per experiment. Scale bars = 500 μm. B, Frequencies of immune cell subsets in spleen were determined by flow cytometry. The gating strategies used to identify the indicated cell subsets are referred to Figure S2A. C, Concentrations of serum immunoglobulin G (IgG) (WT: n = 14, KO: n = 12), IgA (WT: n = 11, KO: n = 11), IgM (WT: n = 21, KO: n = 22), and IgE (WT: n = 10, KO: n = 10) in WT and CPLX2 KO mice were measured by ELISA. D, Serum IgM concentrations were compared between age‐matched male (WT: n = 10, KO: n = 8) and female mice (WT: n = 11, KO: n = 14). A, Data of spleen weight are representative of two independent experiments with two to three mice per experiment. B, Data are pooled from three independent experiments with one to three mice per experiment. C and D, Data are pooled from four independent experiments with two to six mice per experiment. Data are presented as mean ± SEM. CPLX, complexin; ELISA, enzyme‐linked immunosorbent assay; H&E, hematoxylin & eosin; KO, knockout; N.D., not detected; WT, wild‐type. ****P < .0001 and ***P < .001 compared with WT (Mann‐Whitney *U* test)

As immunophenotypic analysis has not been reported in CPLX2 KO mice, we examined the size and histology of mouse spleens and identified immune cell subsets by flow cytometry. There was no difference in the weight, histological analysis (Figure 2A), and immune cell subsets (Figure 2B) of spleen cells between wild‐type (WT) and CPLX2 KO mice.

We further measured the serum levels of total IgG, IgA, IgM, and IgE in naïve WT and CPLX2 KO mice. The level of IgM in CPLX2 KO mice was higher than that in WT with no differences observed for the other immunoglobulin isotypes (Figure 2C). It is well‐known that males and females differ in their immunological responses (eg, serum immunoglobulin levels). Therefore, serum IgM concentrations were compared between age‐matched male and female mice (Figure 2D). IgM concentrations in CPLX2 KO mice were higher than those in WT mice. This is true for both males and females.

We compared the level of four sub‐isotypes of IgG: IgG1, IgG2b, IgG2c, and IgG3. We found no significant differences between WT and CPLX2 KO mice (Figure S1A).

### 3.3 Frequencies of B‐cell subpopulations are similar between CPLX2 KO and WT mice

**Figure 3:**
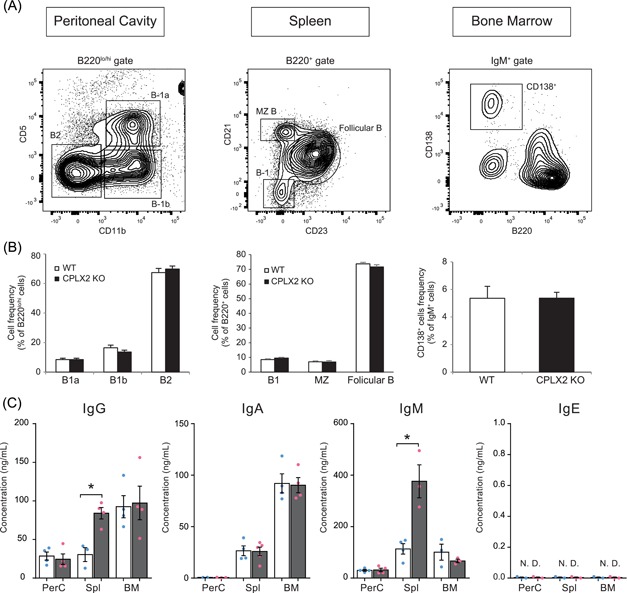
nIgM secretion was enhanced in splenic B cells of CPLX2 KO mice. A, Gating strategy used to define B‐cell subpopulations. Representative plots of B‐cell subpopulations of PerC, spleen, and BM. Gate on B220^lo/hi^, B220^+^, and IgM^+^ cells are shown in the plot of PerC, spleen, and BM. B, The percentages of B‐cell subpopulations in PerC (n = 10), spleen (n = 10), and BM (n = 4) were determined. C, PerC, spleen, and BM cells isolated from WT and CPLX2 KO mice were cultured without stimulation. After 16 hours, culture supernatants were collected, and levels of antibodies were assessed using ELISA. A, One representative example from data in B. B, Data are pooled from two independent experiments with two to four mice per experiment. C, Data are pooled from two independent experiments with two mice per experiment. The data are presented as mean ± SEM from four independent experiments. BM, bone marrow; CPLX, complexins; ELISA, enzyme‐linked immunosorbent assay; KO, knockout; nIgM, natural immunoglobulin M; N.D., not detected; PerC, peritoneal cavity; WT, wild‐type. *P ≤ .05 compared with WT (Mann‐Whitney *U*‐test)

Natural IgM (nIgM)‐secreting cells are known to exist in PerC, spleen, and bone marrow (BM).^24^ PerC B‐1 cells that secrete low levels of nIgM spontaneously^25^ are divided into B‐1a and B‐1b cells (Figure 3A). The primary reported sources of nIgM in serum are B‐1^26^ and marginal zone (MZ) B cells of the spleen and IgM^+^CD138^+^ cells of BM (Figure 3A).^27^ To evaluate the correlation between high levels of serum nIgM and numbers of nIgM‐secreting cells in CPLX2 KO mice, we compared the frequency of nIgM‐secreting cells by analyzing the frequency of B‐1 cells by separating the B220^+^ spleen cells into CD21^hi^/CD23^−^ MZ B and CD21^lo^/CD23^−^ cells in CPLX2 KO and WT mice and found no significant difference in any nIgM‐secreting subpopulation (Figure 3B). Thus, the higher level of serum nIgM in CPLX2 KO mice cannot be explained by an increase in the number of nIgM‐secreting cells based on this analysis. Additionally, there was no significant difference between CPLX2 KO and WT mice in the frequency of other B‐cell subpopulations (PerC B2 cells and follicular B cells) that secrete antigen‐specific antibodies via T–cell‐dependent pathway (Figure 3B).

### 3.4 CPLX2 is involved in natural IgM secretion by splenic antibody‐secreting cells

We assessed the levels of spontaneous IgG, IgA, IgM, and IgE secretion in culture supernatants from PerC, spleen, and BM cells without stimulation. We detected higher levels of IgM only in supernatants from CPLX2 KO splenic cells (Figure 3C). Surprisingly, we found that, compared with WT, the CPLX2 KO splenic cells secreted higher levels of total IgG (Figure 3C). We next measured the levels of the four IgG subisotypes secreted by splenic cells and found that the secreted IgG1 level from CPLX2 KO cells was significantly higher than that from WT cells (Figure S1B). It is reported that mouse splenic plasmablasts, generated from MZ B cells, secrete not only nIgM via T–cell‐independent pathway but also low‐affinity IgG1 via T–cell‐dependent pathway^28^ spontaneously.

## 4 DISCUSSION

Neurotransmitters are secreted by exocytosis of synaptic vesicles from the plasma membrane induced by the assembly of SNARE complex.^2^ CPLXs bind to the SNARE complex and prevent spontaneous vesicle fusion.^10^ It was shown that spontaneous secretion is enhanced in the absence of CPLX.^11^ Similar to its function in the neuronal synapse, we hypothesize that CPLXs in lymphocytes may associate with the membrane fusion machinery. In this study, we found that CPLX2 was expressed only in B cells and not in T cells. Since B cells secrete immunoglobulins spontaneously, our findings raise the possibility that CPLX2 controls spontaneous vesicle fusion and regulates the secretion of antibody‐secreting cells (ASCs), like neuronal synapses. To clarify the mechanisms of secretory processes in B and T cells, it is necessary to examine the expression of all SNAREs that have not been reported in immune cells.

The serum level of IgM in naive CPLX2 KO mice was higher than that in WT, with no difference observed for the other immunoglobulin isotypes. Of the four isotypes of immunoglobulin, IgM is the first antibody to be produced in the humoral immune response and is considered a natural antibody. While nIgM antibodies are secreted constitutively and play a role in preventing infection and maintaining homeostasis, large amounts of nIgM are secreted rapidly in response to microbial infection.^29^ Our new finding that serum nIgM is increased in CPLX2 KO mice indicates that CPLX2 has a regulatory role in the secretion of nIgM. It is considered that the major source of natural antibody in serum are B‐1^26^ and MZ B cells of spleen.^25^ Furthermore, the level of IgM in the culture supernatants of splenic cells from CPLX2 KO mice was higher than that from WT mice. Therefore, we think that one of the CPLX2 roles is a regulation of constant IgM secretion in spleen B‐1 cells and MZ B cells. This study assumes that CPLX2 regulates B‐1 and MZ B cells to secrete IgM constantly. Since there is a possibility that CPLX2 regulates antibody secretion of other ASCs, clarification is needed on the effect of CPLX2 on antibody secretion in stimulated conditions. Further analysis is also necessary to elucidate the role of CPLX2 in T–cell‐dependent antibody secretion.

Here, we report for the first time that CPLX2 is expressed in B cells and acts as an inhibitor of spontaneous secretion of nIgM and IgG1 from splenic ASCs. Further exploration of the molecular mechanism regulating spontaneous vesicle fusion by CPLX2 may lead to an understanding of diseases caused by abnormal secretion of antibodies, such as autoimmune diseases.

## CONFLICT OF INTERESTS

The authors declare that there are no conflict of interests.

## AUTHOR CONTRIBUTIONS

ET and MT designed research. ET and KO were responsible for data collection and data analysis. ET, KS, MN, and MT contributed to the writing and reviewing the final manuscript.

## ETHICS STATEMENT

All animal experiments were performed in accordance with the Regulation for Animal Experiments at Kochi University and were approved by the Kochi University Animal Care and Use Committee (license L‐024).

## Supporting information

Supporting information

Supporting information

Supporting information

Supporting information

## Data Availability

All the data presented here are new and fully accessible.
