# Supplementary material for: Complexin 2 regulates secretion of immunoglobulin in antibody‐secreting cells
Source: Immun Inflamm Dis. 2019 Nov 5;7(4):318–25. doi: 10.1002/iid3.276 (PMC6842823; doi:10.1002/iid3.276)
Supplement: Supplementary file 4 — Supporting information [file IID3-7-318-s004.docx]

## **Supporting Information Figure 1. Levels of four IgG isotypes of WT and CPLX2 mice.**

(A) Concentration of serum IgG1 (WT: n = 4, KO: n = 4), IgG2b (WT: n = 14, KO: n = 14), IgG2c (WT: n = 4, KO: n = 7), and IgG3 (WT: n = 4, KO: n = 4)) in WT and CPLX2 KO mice measured by ELISA. (B) Splenocytes isolated from WT and CPLX2 KO mice were cultured without stimulation. After 16 h, culture supernatants were collected, and levels of antibodies were assessed using ELISA. A: data are pooled from three independent experiments with two to five mice per experiment. B: data are pooled from two independent experiments with two mice per experiment. The data are presented as mean ± SEM from four independent experiments. **p* ≤ 0.05 compared with WT (Mann-Whitney *U*-test).

## **Supporting Information Figure 2. Full gating strategy in flow cytometry analysis.**

(A) Gating strategy used to define leukocyte subsets. Whole blood cells were gated on forward scatter (FSC)/side scatter (SSC) plot. Leukocyte subsets were further gated to B cell, T cell, natural killer (NK), natural killer T (NKT), neutrophil, and monocyte. (B) Gating strategy used to define B cell subpopulations. Whole blood cells were gated on FSC/SSC plot. Subpopulations of PerC were further gated on B220^lo/hi^ to identify B-1a, B-1b, and B2. Subpopulations of spleen were further gated on B220^+^ to identify MZ B and follicular B cells. Subpopulations of BM were further gated on IgM^+^ to identify CD138^+^ IgM-secreting cells.

## **SUPPORTING INFORMATION**

## Additional supporting information may be found online in the Supporting Information, including: Levels of four IgG isotypes of WT and CPLX2 KO mice (Figure S1), Full gating strategy in flow cytometry analysis (Figure S2), and Primers used for RT-PCR (Table 1).
